# Exploring core and bridge symptoms in patients recovering from stroke: a network analysis

**DOI:** 10.3389/fneur.2024.1434303

**Published:** 2024-10-02

**Authors:** Yao Huang, Songmei Cao, Teng Li, Jingjing Wang, Zhuoran Xia

**Affiliations:** ^1^Department of Nursing, The Affiliated Hospital of Jiangsu University, Zhenjiang, China; ^2^Department of Neurology, Changzhou Seventh People’s Hospital, Changzhou, China

**Keywords:** stroke, convalescence, symptom, China, patient

## Abstract

**Background:**

Patients recovering from stroke experience a variety of symptoms that present as a synergistic and mutually reinforcing “symptom cluster,” rather than as singular symptoms. In this study, we researched and systematic analyzed these symptom clusters, including core and bridge symptoms, to help determine the relationships between symptoms and to identify key symptom targets, providing a new approach for formulating precise symptom management interventions.

**Methods:**

Convenience sampling was applied to select 432 stroke recovery patients treated in the Seventh People’s Hospital of Changzhou City from August 1, 2023 to April 14, 2024. Subsequently, a cross-sectional survey was conducted using the General Information Questionnaire and Stroke Symptom Experience Scale to extract symptom clusters via exploratory factor analysis. Finally, the “qgraph” and “bootnet” packages in the R language were used to construct a network layout to describe the relationships between symptoms and calculate the centrality index.

**Results:**

The average age of the 432 enrolled recovering stroke patients was 68.17 ± 12.14 years, including 268 males (62.04%) and 164 females (37.96%), none of whom underwent surgical intervention. Among this cohort, the 3 symptoms with the highest incidence rates were “limb weakness” (A2, 80.56%), “fatigue” (A5, 77.78%), and “limitations of limb movement” (A1, 68.06%). A total of 5 symptom clusters were extracted: the somatic activity disorder, mood-disorder-related, cognitive–linguistic dysfunction, somatic-pain-related, and foot dysfunction symptom clusters. In the symptom network, the 2 most common symptoms in terms of intensity and expected impact were “fatigue” (A5, r_s_ = 1.14, r_e_ = 1.00) and “pessimism about the future” (B3, r_s_ = 1.09, r_e_ = 1.02). The symptom with the strongest bridge intensity was “limb pain” (D1, r_s_ = 2.64).

**Conclusion:**

This study uses symptom network analysis to explore the symptoms of stroke patients during recovery, identifying core symptoms and bridge symptoms. Based on these findings, we can develop more targeted management plans to improve the accuracy and efficiency of interventions. Through this management approach, we can enhance treatment effectiveness, reduce unnecessary medication, lower adverse drug reactions, and optimize the allocation of medical resources.

## 1 Introduction

Stroke is an acute cerebrovascular disease caused by the sudden rupture of blood vessels in the brain, or the obstruction of blood vessels, resulting in a lack of blood flow to the brain, resulting in brain tissue damage or dysfunction. This disease is characterized by high prevalence, mortality, recurrence, and disability rates (1, 2). According to Global Burden of Disease study data (3), as of 2019, there were approximately 101 million stroke patients worldwide, with stroke emerging as the second leading cause of death worldwide. As the emergency medical service system has continuously improved, the survival rate of stroke patients has improved; however, after acute treatment, these patients need to progress through a long recovery. Patients in the poststroke recovery period not only suffer from complications caused by the disease itself, such as hemiparesis, aphasia, and dysphagia, but are also prone to symptoms such as depression, anxiety, fatigue, sleep disorders, and chronic pain (4–7). These symptoms often co-occur and are interrelated, with varying degrees of severity, forming distinct symptom clusters. Synergistic effects between symptom clusters can further aggravate the symptom burden of patients, seriously impacting their quality of life (8).

A review of the prior literature on post-stroke sequelae revealed that many studies primarily focused on exploring single symptoms experienced by stroke patients, such as fatigue (8), sleep disturbances (9), depression (10), and anxiety (9), with less attention paid to symptom clusters, core symptoms, and bridging symptoms. For example, Schepers et al. (11) predicted the occurrence of depressive symptoms in recovering stroke patients in a longitudinal follow-up study; while Kirkevold et al. (12) explored the experience, prevalence, characteristics, and contributing factors of fatigue poststroke in a qualitative interview study. Further, using a questionnaire-based study, Wallace et al. (13) reported that stroke may further exacerbate sleep disturbances, which can in turn affect the stroke recovery process and increase the risk of stroke recurrence. Diamond et al. (14) explored the prevalence of anxiety in stroke survivors and its relative impact on quality of life following a cross-sectional study design. Although useful, all of these prior studies explored various sequelae caused by stroke, but subsequently focused on only one symptom. In fact, patients who have recovered from stroke rarely experience only a single symptom, and the common interaction of multiple symptoms may increase the symptom burden of patients recovering from stroke, leading not only to impaired physical functioning, but also seriously impacting their psychological and social functioning. However, few studies have provided information about symptom clusters in patients recovering from stroke, which is crucial for improving the efficacy of symptom interventions (8–14). As such, the present study aimed to provide information about symptom clusters in patients recovering from stroke, which is crucial for improving the efficacy of symptom interventions.

Although the concept of symptom clusters can facilitate cluster symptom management, the lack of differentiation between primary and secondary relationships can lead to ineffective management. In the context of relationships, the terms “primary” and “secondary” are commonly used to describe the level of importance or significance. Primary relationships are typically the most important related core symptom (15), while secondary relationships include relationships that are still important and meaningful, but may not carry the same level of importance in stroke patients as primary relationships (16). In recent years, the concept of symptom networks has been gradually applied in chronic disease management, an approach that uses nodes and edges to represent symptoms and their relationships, providing a new method to identify core symptoms and gain insight into the complexity of symptom clusters by visualizing and quantitatively interpreting the relationships between various symptoms and symptom clusters (17). The core symptoms in a network include those that are most strongly associated with other symptoms, playing a key role in activating other symptoms (18). Interventions targeting core symptoms can accelerate the deactivation of the symptom network, as well as increasing the precision and efficiency of interventions (19, 20). In addition, previous related studies (21–23) have shown that bridging symptoms are associated with the structure of symptom clusters in the symptom network. Bridge nodes or edges play a critical transmission role and accelerate the spread of information in the propagation of symptoms from one cluster to another (24). By intervening in bridging symptoms, we can prevent the interconversion of symptoms, thereby breaking the connections between symptom clusters and reducing the symptom burden faced by patients.

The primary objectives of this study were to identify symptom occurrence and analyze symptom clusters in patients recovering from stroke and to generate a symptom network of patients recovering from stroke, exploring core and bridge symptoms to provide a basis for symptom management in patients recovering from stroke.

## 2 Materials and methods

### 2.1 Study design and participants

**Figure 1:**
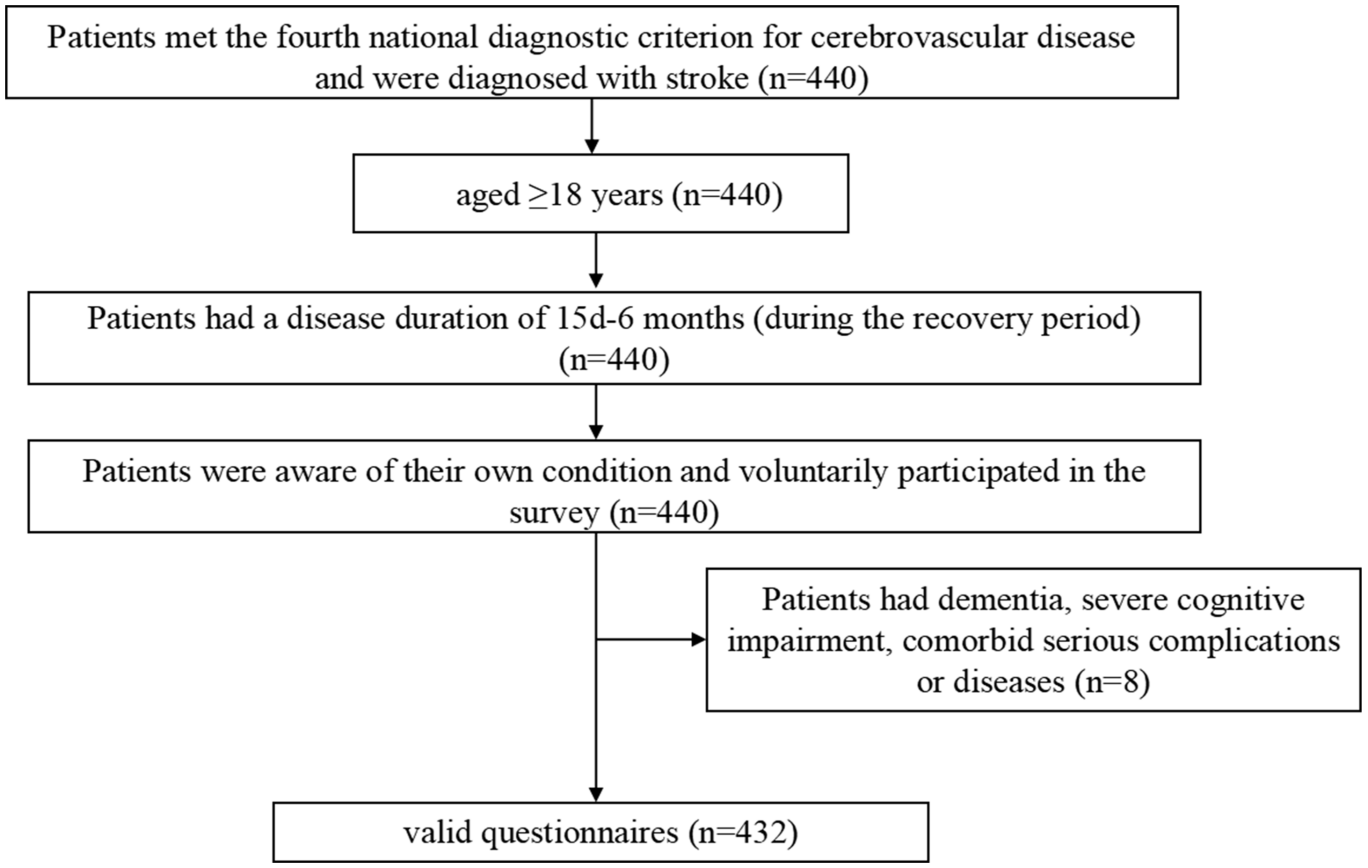
Flowchart of patient inclusion and exclusion.

This was a cross-sectional study designed to investigate the symptom burden of patients recovering from stroke through the identification of relevant symptom clusters, core symptoms, and bridge symptoms. Patients were recruited using convenience sampling from the Seventh People’s Hospital of Changzhou City, Jiangsu Province, China. The inclusion criteria were as follows (Figure 1): (1) age ≥ 18 years; (2) met the fourth national diagnostic criterion for cerebrovascular disease, also known as Diagnostic Criteria of Cerebrovascular Diseases in China (version 2019) (25). This diagnostic point combines the new Chinese version of the Classification of Cerebrovascular Diseases and the International Classification of Diseases-11, emphasizing the role of imaging examinations in diagnosis based on the attention to the symptoms and signs of major types of cerebrovascular diseases; We have refined the etiological diagnostic criteria for various types of cerebrovascular diseases, including the main types of common cerebrovascular diseases in clinical practice. We have provided diagnostic criteria for 11 types of cerebrovascular diseases including Ischemic cerebrovascular disease, hemorrhagic cerebrovascular disease, head and neck, atherosclerotic stenosis or occlusion (not cerebral infarction), hyperemia pressure encephalopathy, primary central nervous system vasculitis, other cerebrovascular diseases disease, intracranial venous thrombosis, no acute focal neuropathy functional impairment of cerebrovascular disease, post-stroke sequelae, and vascular cognition obstacles and post-stroke emotional disorders, and further subdivided them into important subtypes based on lesion location, pathology, and etiology. For some special types of clinical processes, diagnostic criteria have also been proposed. It is the most specific, comprehensive, and accurate diagnostic key for cerebrovascular disease in China as a guide for clinical doctors in diagnosis, treatment, and management over the years. And were diagnosed with stroke confirmed by Tomography, X-Ray Computed (CT) or Magnetic Resonance Imaging (MRI) (26); (3) had a disease duration of 15 days-6 months during the recovery period; (4) were aware of their own condition; and (5) voluntarily participated in the survey. The exclusion criteria were as follows: (1) diagnosis of dementia or severe cognitive impairment (27); (2) serious mental illness (28); (3) coexisting with other serious illnesses such as other neurological disorders, acute infections and hematopoietic dysfunction; (4) short expected survival period; (5) recent history of major surgeries or injuries; (6) drug or alcohol dependence; and (7) or unable to cooperate with the evaluation. The patient was treated from August 1, 2023 to April 14, 2024 at the inpatient department of neurology and neurosurgery of Changzhou Seventh People’s Hospital. The study was approved by the Ethics Committee of the Seventh People’s Hospital of Changzhou City (approval number: LC2024001).

The investigators were contacted by the researcher together with 2 undergraduate nursing students who received uniform training. Stroke patients who met the criteria were screened and informed of the purpose and content of the study, as well as the anonymous, confidential, and voluntary nature of the survey. Subsequently, informed consent was obtained from the patients by signing an informed consent form in writing. All surveys were conducted by the investigator using face-to-face interviews who asked the respondents all of the questions, filled out the questionnaire according to the respondents’ answers, and indicated “unknown” or “refused” if the respondents were unwilling or unable to answer. At the end of the survey, the investigator checked the completeness of the questionnaire and thanked the respondents and their families. As the data for this study were collected independently by multiple investigators, we further conducted telephone callbacks to clarify questionable responses during the data compilation process to verify the information. The validity and reliability of the data were thus ensured.

### 2.2 Measurements

#### 2.2.1 Demographic information and disease characteristics

Socio-demographic information includes age, gender, body mass index (BMI), literacy, medical payment methods, occupational status, and nature of the primary caregiver. Among these, the BMI was calculated from each individual’s weight and height. According to the standards set by the World Health Organization, BMI is classified as follows: underweight (BMI < 18.5), normal weight (18.5 ≤ BMI < 24.9), overweight (25 ≤ BMI < 29.9), and obesity (BMI ≥ 30). Literacy refers to the level of education an individual had received, classified as no education, secondary school, junior high school, high school, and university and above. Medical payment methods refer to the ways in which an individual or family pays for healthcare services, which was classified three main types: employee medical insurance, resident medical insurance, and out-of-pocket payments. Employee medical insurance applied to urban workers and was funded by both the employer and the employee, offering a higher reimbursement rate; resident medical insurance applied to urban residents without employee medical insurance and provided a lower reimbursement rate; and out-of-pocket refers to the full payment of medical expenses by the individual due to the absence of medical insurance. Occupational status includes categories such as retired (from work), laid off, on duty, and self-employed. The primary caregiver was defined as the person providing primary support and care for the patient, whether medically or in daily life; this could be a spouse, child, parent, sibling, babysitter, or nanny.

The Disease Characterization Questionnaire includes details such as post-stroke duration, comorbid chronic diseases, control of chronic diseases, type of stroke, number of disease episodes, and receipt of rehabilitation exercise therapy. Post-stroke duration was categorized as period from 15 days to 1 month after the stroke, 3 months after the stroke, and 6 months after the stroke. Sufficient control of chronic diseases refers to the appropriate treatment and management to maintain a patient’s physiological indicators, such as blood sugar, blood pressure, and cholesterol, within a reasonable range, while also improving symptoms. Rehabilitation via exercise treatment refers to the participation of patients in personalized rehabilitation programs at rehabilitation facilities to promote the recovery of physical function and enhance overall health.

#### 2.2.2 Stroke symptom experience scale

The Stroke Symptom Experience Scale developed by Shi et al. (29) in 2019, which was self-designed using the Symptom Experience Model as a theoretical framework, was used as the symptom assessment scale. This scale had 19 symptom entries: limitation of limb movement, limb weakness, limb pain, shoulder pain, foot drop, foot inversion (pathological), uncoordinated limb movements, inability to maintain balance, memory loss, poor concentration, delayed reaction time, difficulty speaking, easily anxious, sullen, pessimism about the future, disinterest in surroundings, distress from an inability to engage in desired activities, fatigue, and decreased self-care ability. The terms “limb weakness” and “limitation of limb movement” describe distinct phenomena that can have different underlying causes and require different management strategies. “Limb weakness” refers to a reduced ability to generate force with a limb, which commonly manifests as difficulty in lifting, pushing, or pulling objects (30). This can be caused by a variety of factors, including muscle atrophy, nerve damage, or neuromuscular disorders. In some cases, limb weakness may be accompanied by a limitation of limb movement, but this is not always the case. Conversely, “limitation of limb movement” refers to a restriction in the range of motion of a limb, which can be caused by factors such as joint stiffness, pain, or muscle contractures (31). This limitation can occur without any underlying weakness in the limb, and can significantly impact an individual’s ability to perform daily tasks. The presence or absence of these symptoms was determined during symptom assessment; if symptoms were present the frequency, intensity, and degree of distress were further evaluated. Frequency and intensity were scored from 1 to 4, with 1 indicating mild, 2 moderate, 3 severe, and 4 very severe; distress was scored from 0 to 4, with 0 indicating none at all, 1 a little, 2 some, 3 more, and 4 a lot. The mean value of the scores of the three dimensions of the symptom indicates the patients’ symptom burden, for which a higher score indicates a stronger symptom burden. The mean content validity index of the scale amounted to 0.947, while the content validity indices of the entries ranged from 0.800 to 1.00. In addition, the correlation coefficient between the scale and the Stroke Impact Scale was −0.714, indicating a good correlation. The internal consistency of the scale was measured as having a Cronbach’s alpha coefficient of 0.810, and the Cronbach’s alpha coefficients for the dimensions of physical symptoms and self-care decline, cognitive decline, and psychological symptoms were 0.759, 0.859, and 0.730, respectively. In addition, the scale’s folded-half reliability was 0.760, further supporting its reliability.

### 2.3 Statistical analysis

#### 2.3.1 Analysis of symptom cluster types

Excel 2019 was used for data entry. In the descriptive analysis, normally distributed continuous variables were described as the mean ± standard deviation. Continuous variables with skewed distributions were described using median and quartiles, with means as the auxiliary evaluation data. Categorical variables were described as the frequency and percentage. The severity scores of symptoms with an incidence of ≥25% were analyzed using exploratory factor analysis to identify symptom clusters of patients recovering from stroke. After extracting the symptom clusters, the included symptoms were subjected to network analysis.

#### 2.3.2 Symptom network

We constructed an undirected network model containing all 19 symptoms using the R package “qgraph”. In the symptom network, each node represents a single symptom, while the edge between two nodes represents the conditional independent relationship between two symptoms. The thicker the edge is, the stronger the correlation between the two symptoms (20). The Fruchterman–Reingold algorithm was applied to visualize the network, with the most strongly correlated nodes located in the centre of the network, nodes with similar characteristics located relatively close to each other, and nodes with weaker and fewer connections located at the periphery of the symptom network.

#### 2.3.3 Centrality and bridge centrality

We used the R package “qgraph” for centrality analysis. The centrality metrics of the computational model included strength; defined as the sum of direct connections between symptoms, used to assess the importance of symptoms; and expected impact, used to measure the influence of symptoms in the network and is one of the most reliable centrality metrics for determining the between-symptom relationships (32). Bridge strength has been reported as the best bridge centrality metric for identifying nodes, and is used to identify important symptoms that connect different subnetworks (24). Bridge symptoms are widely defined as symptoms that connect different symptom clusters (23). The R package “networktools” was applied to identify bridging symptoms and assess bridge strength between clusters.

#### 2.3.4 Accuracy, reliability and difference tests

The 95% confidence intervals associated with the bootstrapped edge weights were applied to estimate the accuracy of the network edges. The reliability of the centrality metrics occurring after network sample-size reduction were tested using the R package “bootnet,” while the correlation reliability was considered to be indicated by correlation reliability coefficients not lower than 0.25 and preferably higher than 0.50 (33). Regularization based on a graphical lasso (glasso) partial correlation matrix was applied to test the bootstrap variability of the edge weights and intensity centrality metrics.

## 3 Results

### 3.1 General information of the study population

A total of 440 questionnaires were distributed, from which 432 valid questionnaires were obtained, yielding a questionnaire recovery validity rate of 98.2%. The average age of the study subjects was 68.17 ± 12.14 years; 73 patients (16.9%) had hemorrhagic strokes, and 395 patients (83.1%) had ischemic strokes. The other general information on the study subjects is displayed in Table 1.

**Table 1:** General information of the study population (*n* = 432).

Variable		Number of patients (*n* (%))
Age	<60 years	110 (25.5)
	≥60 years	322 (74.5)
Sex	Male	268 (62.0)
	Female	164 (38.0)
Body mass index (BMI)	Underweight	16 (3.7)
	Normal weight	211 (48.8)
	Overweight	151 (35.0)
	Obese	54 (12.5)
Educational attainment	No education	25 (5.8)
	Secondary school	247 (57.2)
	Junior high school	104 (24.1)
	High school and secondary school	30 (6.9)
	University and above	26 (6.0)
Medical payment methods	Employee medical insurance	214 (49.5)
	Resident medical insurance	186 (43.1)
	Out of pocket	32 (7.4)
Occupational status	Retired (from work)	272 (63.0)
	Laid off	21 (4.9)
	On duty	82 (18.9)
	Self-employed	57 (13.2)
Primary caregiver	Spouse	210 (48.6)
	Children	187 (43.3)
	Parents, siblings, etc.	16 (3.7)
	Nannies	19 (4.4)
Post-stroke duration	15 days to 1 month after stroke	152(35.2)
	3 months after stroke	83(19.2)
	6 months after stroke	197(45.6)
Comorbidity with other chronic diseases	Yes	350 (81.0)
	No	82 (19.0)
Sufficient control of chronic diseases	Yes	261 (60.4)
	No	171 (39.6)
Types of stroke	Ischemic stroke	359 (83.1)
	Hemorrhagic stroke	73 (16.9)
Number of disease episodes	One	295 (68.3)
	Two or more	137 (31.7)
Underwent rehabilitation via exercise treatment	Never	220 (50.9)
	Infrequent	94 (21.8)
	Nonrecurrent	118 (27.3)

### 3.2 Occurrence of symptoms in patients recovering from stroke

As shown in Table 2, the primary symptoms experienced by the enrolled participants were counted and summarized, and included limitation of limb movement, Limb weakness, Foot drop, etc. The top 3 symptom incidence rates in this study were found for “limb weakness” (A2, 80.56%), “fatigue” (A5, 77.78%), and “limitation of limb movement” (A1, 68.06%); the top 3 symptom severity rates were found for “limb weakness” (A2), “fatigue” (A5), and “limitation of limb movement” (A1).

**Table 2:** Incidence and severity of symptoms among patients who recovered from stroke (*n* = 432).

Symptom	Number of patients (*n* (%))	Severity	
		M (P25, P75)	Average
limitation of limb movement (A1)	294 (68.06)	2 (0, 3)	1.67
Limb weakness (A2)	348 (80.56)	2 (1, 3)	2.01
Limb pain (D1)	110 (25.46)	0 (0, 1)	0.57
Shoulder pain (D2)	24 (5.56)	0 (0, 0)	0.11
Foot drop (E1)	30 (6.94)	0 (0, 0)	0.11
Foot inversion (pathological) (E2)	22 (5.09)	0 (0, 0)	0.10
Uncoordinated limb movements (A3)	178 (41.20)	0 (0, 3)	1.13
Inability to maintain balance (A4)	197 (45.60)	0 (0, 3)	1.18
Memory loss (C1)	131 (30.32)	0 (0, 1)	0.67
Poor concentration (C2)	139 (32.18)	0 (0, 1)	0.65
Delayed reaction time (C3)	180 (41.67)	0 (0, 1)	0.80
Difficulty speaking (C4)	183 (42.36)	0 (0, 2)	1.02
Easily anxious (B1)	111 (25.69)	0 (0, 0)	0.60
Sullen (B2)	110 (25.46)	0 (0, 0)	0.56
Pessimism about the future (B3)	95 (21.99)	0 (0, 0)	0.53
Disinterest in surroundings (B4)	89 (20.60)	0 (0, 0)	0.51
Distress from inability to engage in desired activities (B5)	82 (18.98)	0 (0, 0)	0.49
Fatigue (A5)	336 (77.78)	2 (1, 3)	1.82
Decreased self-care ability (A6)	274 (63.43)	1 (0, 3)	1.51

### 3.3 Extraction of symptom clusters during stroke recovery

Nineteen symptoms were included in this study for factor analysis, for which the Kaiser–Meyer–Olkin (KMO) value in factor analysis was 0.818, and that Bartlett’s spherical test result was X^2^ = 2691.717 (*p* < 0.01), indicating suitability for factor analysis. Table 3 presents the results of exploratory factor analysis of symptom clusters in patients who recovered from stroke. A total of five common factors were extracted in this analysis, yielding a cumulative variance contribution rate of 68.131%. The five clusters were named according to the characteristics of the symptoms in each cluster: the somatic activity disorder, mood-disorder-related, cognitive–linguistic dysfunction, somatic-pain-related, and foot dysfunction symptom clusters.

**Table 3:** Factor loadings for symptoms in patients recovering from stroke.

Symptomatic	Factor 1	Factor 2	Factor 3	Factor 4	Factor 5
**Somatic activity disorder symptom cluster**					
Limb weakness (A2)	0.883				
limitation of limb movement (A1)	0.826				
Decreased self-care ability (A6)	0.814				
Inability to maintain balance (A4)	0.803				
Uncoordinated limb movements (A3)	0.777				
Fatigue (A5)	0.485				
**Mood-disorder-related symptom cluster**					
Disinterest in surroundings (B4)		0.899			
Pessimism about the future (B3)		0.882			
Distress from inability to engage in desired activities (B5)		0.878			
Sullen (B2)		0.850			
Easily anxious (B1)		0.630			
**Cognitive–linguistic dysfunction symptom cluster**					
Delayed reaction time (C3)			0.802		
Memory loss (C1)			0.784		
Poor concentration (C2)			0.746		
Difficulty speaking (C4)			0.623		
**Somatic-pain-related symptom cluster**					
Limb pain (D1)				0.860	
Shoulder pain (D2)				0.629	
Foot dysfunction symptom cluster					
Foot inversion (pathological) (E2)					0.804
Foot drop (E1)					0.598
Eigenvalue (math.)	3.902	3.864	2.456	1.470	1.253
Variance contribution (%)	20.539	20.338	12.925	7.735	6.594
Cumulative variance contribution (%)	20.539	40.877	53.802	61.537	68.131

### 3.4 Network analysis of symptoms in patients recovering from stroke

**Figure 2:**
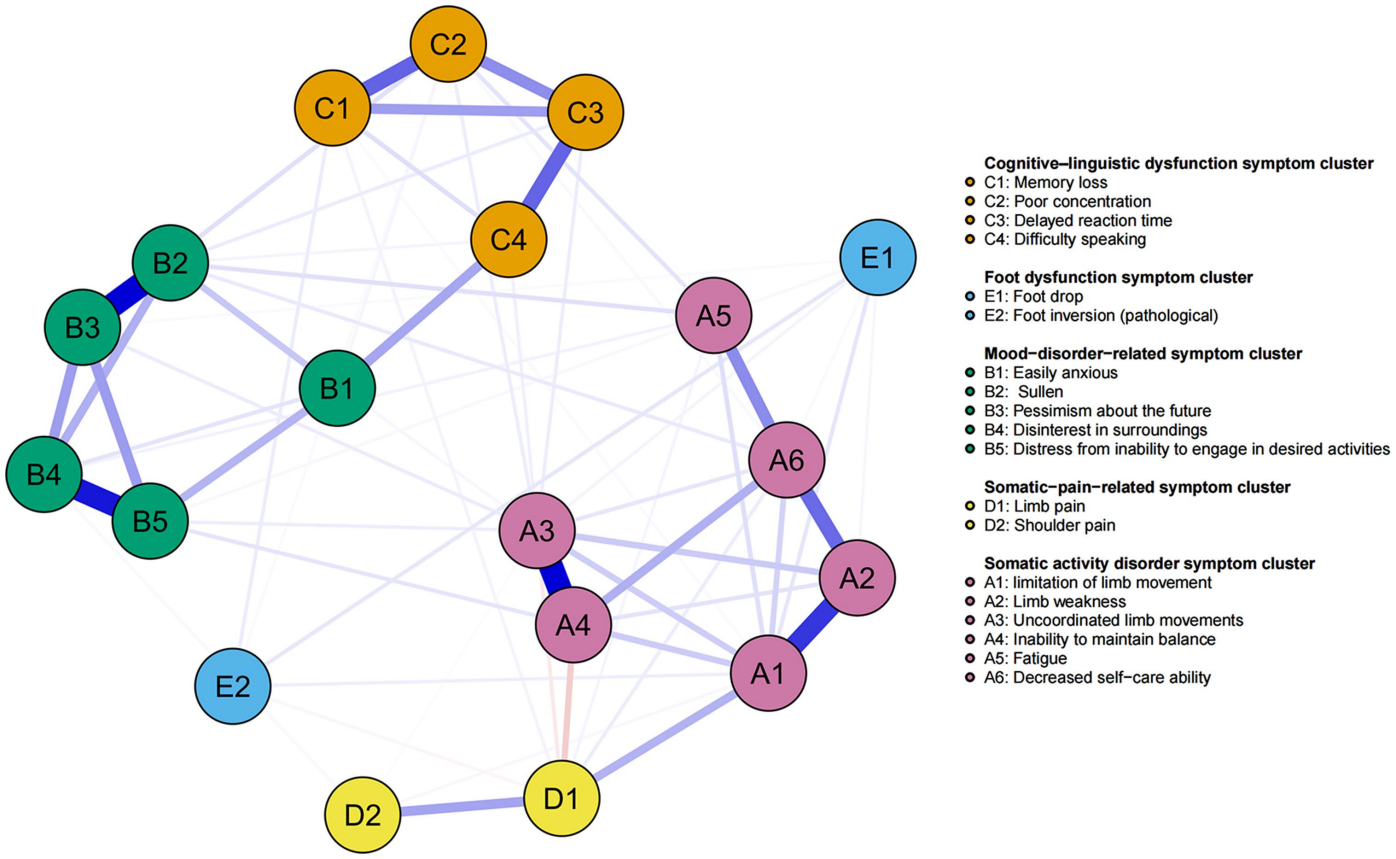
Symptom network of patients recovering from stroke. The five different colors represent the five different symptom clusters. Blue and red edges represent positive and negative correlations, respectively. The thickness of the edges represents the strength of the link between the symptoms.

**Figure 3:**
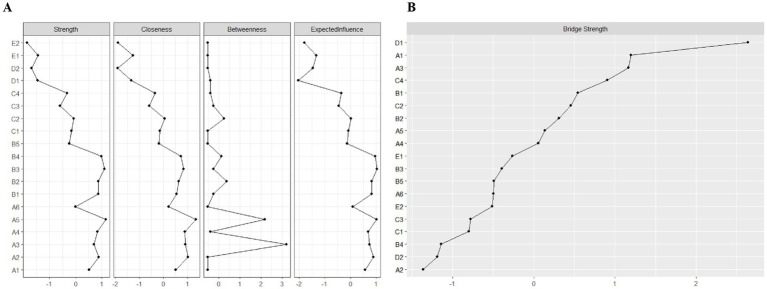
Indicators of the centrality of symptom network nodes, including: **(A)** Strength, closeness, betweenness, and expected influence centrality; and **(B)** Bridge strength centrality. A1: Limitation of limb movement; A2: Limb weakness; A3: Uncoordinated limb movements; A4: Inability to maintain balance; A5: Fatigue; A6: Decreased self-care ability; B1: Easily anxious; B2: Sullen; B3: Pessimism about the future; B4: Disinterest in surroundings; B5: Distress from inability to engage in desired activities; C1: Memory loss; C2: Poor concentration; C3: Delayed reaction time; C4: Difficulty speaking; D1: Limb pain; D2: Shoulder pain; E1: Foot drop; E2: Foot inversion (pathological).

**Figure 4:**
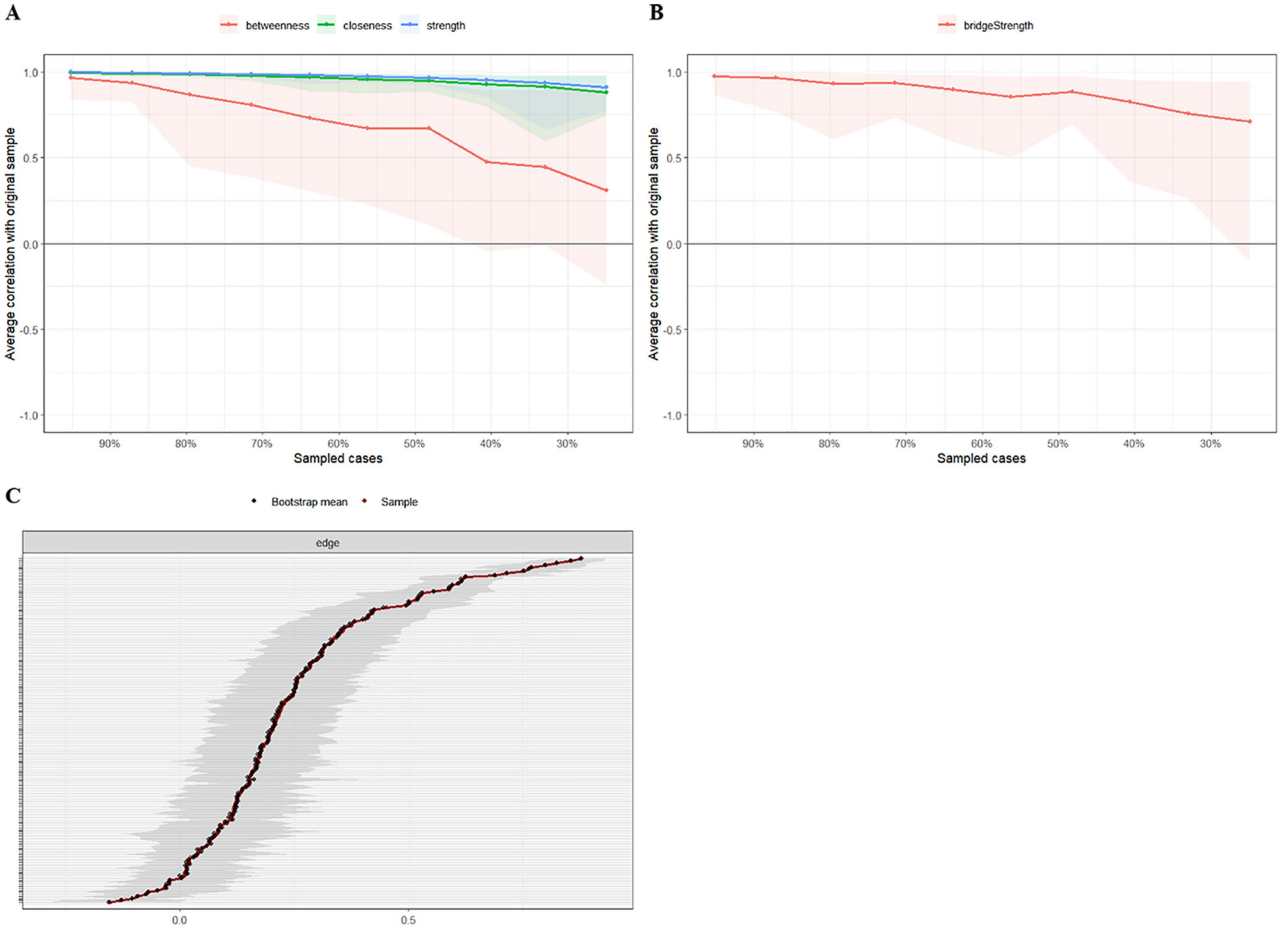
The stability and accuracy of the network structure. **(A)** Strength, closeness, and betweenness stability tests; **(B)** Bridge strength stability tests; **(C)** Accuracy analysis of edge weights.

**Figure 5:**
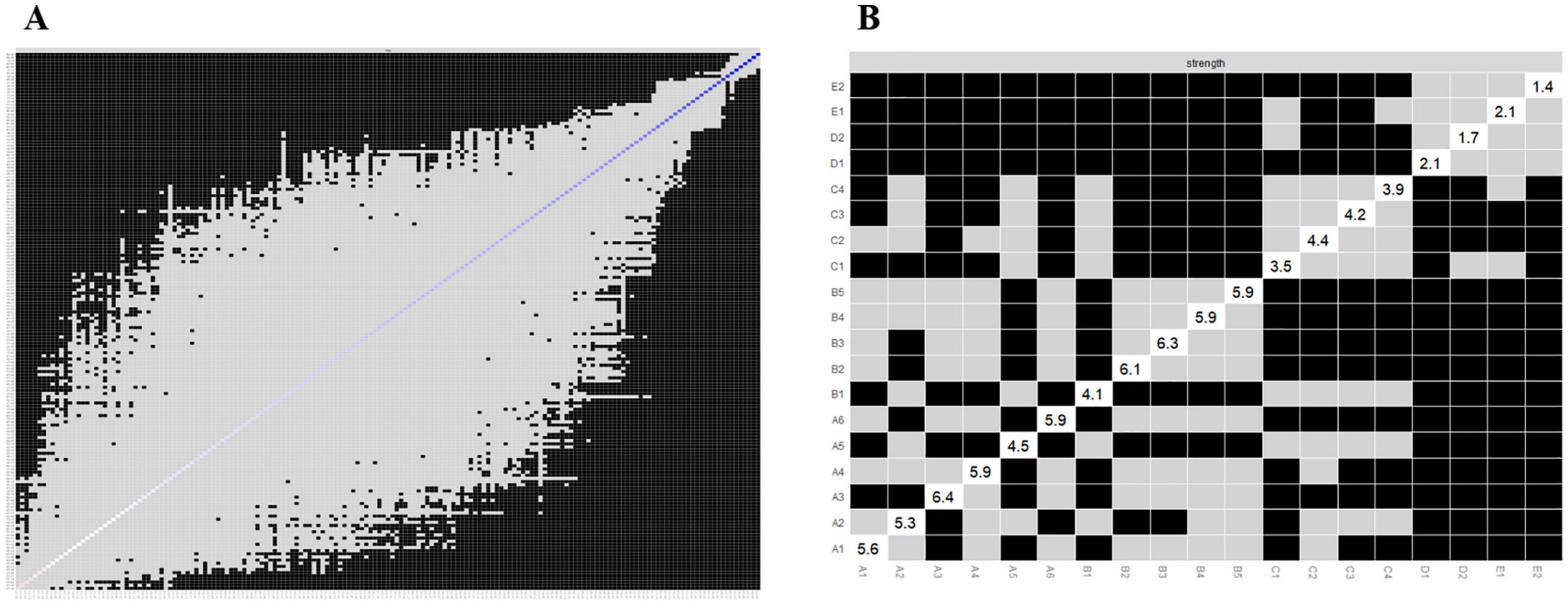
Bootstrapped difference tests for the edge and strength. **(A)** Bootstrapped discrepancy test for edge weighting; **(B)** Bootstrapped discrepancy test for strength centrality.

We applied network analysis to investigate the relationships among 19 common symptoms in patients who recovered from stroke. The edge weights of the symptom network and the results of network analysis (Figure 2) showed that the strongest symptom pairs were “sullenness” and “pessimism about the future” (B2–B3, r = 0.55). According to the node centrality index (Figure 3A), the first ranked in terms of intensity and expected impact were “fatigue” (r_s_ = 1.14, r_e_ = 1.00), followed by “pessimism about the future” (B3, r_s_ = 1.09, r_e_ = 1.02); according to the results of the bridge centrality index (Figure 3B), the symptom with the strongest bridge intensity was “limb pain” (D1, r_s_ = 2.64). The symptom with the strongest bridge intensity according to the centrality index was also “limb pain” (r_s_ = 2.64). The correlation reliability coefficients for strength, tight centrality, mediated centrality, and bridge strength in this study were 0.751, 0.751, 0.205, and 0.518, respectively (Figures 4A,B). The small 95% confidence intervals (grey area) of the edge weights indicate good network precision, as indicated in Figure 4C. Figure 5 presents the results of the bootstrap test of variance, in which black boxes indicate significant differences in the two edge weights of the nodes, or the centrality of each strength (*p* < 0.05).

## 4 Discussion

### 4.1 Symptom clusters in stroke recovery

In this study, we applied exploratory factor analysis to identify 5 symptom clusters among patients recovering from stroke: the somatic activity disorder, mood-disorder-related, cognitive–linguistic dysfunction, somatic-pain-related, and foot dysfunction symptom clusters. Among these, the somatic activity disorder symptom cluster included symptoms of uncoordinated limb movements and decreased self-care ability, which were characterized by a high intensity and inflicted the greatest symptom burden on patients; as such, this symptom cluster was initially recognized as the core symptom cluster in patients recovering from stroke. In support of these results, Huang et al. (34) demonstrated that decreased self-care ability was a core symptom in patients recovering from stroke. Damage to the central nervous system in these patients resulting in weakened or lost control of the motor system and disorganization of reflex movements and intermuscular coordination with prolonged and sustained pulling of the muscles, leads to severe motor impairment in many patients (35). Recent studies have found that the central nervous system, particularly the Guillain-Mollaret triangle, is one of the most important pathways affecting fine motor function recovery (36, 37). The clinical implications of Guillain-Mollaret triangle injuries are profound, as evidenced by the various neurological disorders associated with this region. For example, brainstem cavernous malformations, a rare but serious condition, can affect the Guillain-Mollaret triangle, leading to a constellation of symptoms including the loss of motor control, palatal tremor, and other neurological deficits. Case studies and clinical reports have previously documented the devastating effects of such injuries, emphasizing the need for prompt diagnosis and tailored treatment plans (38). Conversely, one prior study (39) showed that somatic dysfunction affects patients’ daily activities, reduces quality of life, and influences recovery. Yang et al. (40) further concluded that long-term somatic dysfunction not only leads to long-term depression and decreased motivation, but also delays the recovery of neurological functions, such as movement, non-linguistic cognition, and language. As such, we propose that medical professionals should focus on the symptom cluster encompassing patients’ somatic activity disorder, and regularly assess of patients’ somatic function according to their somatic activity disorder.

### 4.2 Core symptoms of stroke recovery

We found that “fatigue” (A5) and “pessimism about the future” (B3), which were identified as the most central symptoms, showed the greatest intensity and expected impact on the network, implying that they play a key role in the stroke symptom network. We also identified “limb weakness” (A2), “fatigue” (A5), and “limitation of limb movement” (A1) as the top 3 symptoms in terms of severity and frequency of occurrence. Indeed, we were surprised to find that the 1st and 3rd symptoms in terms of severity and frequency of occurrence were not symptoms with high centrality. This result indicates that a symptom with a high frequency of occurrence and severity is not necessarily a core symptom, and that it may be affected by other factors, such as the effect of rehabilitation treatment or individual differences in patients. In contrast, fatigue may be more central as it is a prevalent and stable symptom during the recovery period of stroke patients, and may affect the recovery process and quality of life of patients, making them feel more disturbed and frustrated (12). Fatigue is the most central symptom, and prior research (41) has found that approximately half of stroke survivors experience poststroke fatigue, a form of pathologic fatigue characterized by persistent perceptions of physical and mental tiredness (42) that do not improve with rest. Related studies (43) have shown that fatigue in the acute phase of stroke may be related to the severity and location of the lesion, whereas fatigue in the recovery phase is more closely related to psychological (depression and anxiety) and behavioral (coping strategies and inactivity) factors. Patients who have recovered from stroke commonly experience difficulties returning to their premorbid state after treatment and rehabilitation. The prolonged physiological and psychological fallout leads to negative emotions, which in turn affects their recovery and daily activities (12, 44). A recent study by House et al. (45) showed for the first time that fatigue predicts death in the late stage of stroke. However, previous studies (46) have shown that poststroke fatigue is commonly overlooked, that fatigue is not intuitively visible as other symptoms are, while more in-depth knowledge and assessment may be required for fatigue to be recognized and managed. Conversely, effective interventions for poststroke fatigue vary widely, and there are currently no standardized interventions available, which poses a challenge for healthcare professionals (47). In the future, it will be necessary to develop standardized assessment tools to aid healthcare professionals in more accurately identifying and assessing the degree of fatigue in patients so that appropriate treatment measures can be taken. Further, in-depth studies on the effectiveness of measures for managing fatigue symptoms after stroke are needed to provide more individualized and effective treatment plans for patients.

“Pessimism about the future” (B3) was identified as another important core symptom, which indicates the negative emotions that arise in patients recovering from stroke in the face of an unpredictable future. Several relevant studies (48, 49) have shown that this negative emotion is related to the hopes and goals set during the rehabilitation process. False hopes can cause patients to find that the expected recovery outcomes are unattainable, eliciting profound disappointment and frustration, resulting pessimism about the future and disengagement in, or even abandonment of, their rehabilitation efforts. This can exacerbate their physical and mental health problems, thereby creating a cycle of suffering that can exert a sustained negative impact on overall recovery and quality of life (50). Unfortunately, we could not assess the causal relationship between symptoms and recovery. Nevertheless, these results show that pessimism is an important symptom during the recovery period of stroke patients, and needs to be taken seriously. Overall, the setting of realistic goals is crucial in the rehabilitation process, as these goals can be used to provide clear direction and measurable indicators of progress to allow patients to find a balance between hope and the actual likelihood of recovery, and ensure that patients accept their limitations, while maintaining a positive expectation of improvement.

### 4.3 Bridge symptoms in stroke recovery

Bridge symptoms, defined as symptoms that connect different symptom clusters (18), can help to identify interactions between different symptom clusters (51). Marginal weighted analysis showed that “sullen” (B2) and “pessimism about the future” (B3) were most strongly associated. After stroke, patients may require long-term rehabilitation and care, possibly even relying on others for daily living. This decline in quality of life can lead to feelings of frustration and hopelessness, which in turn may lead to pessimism about the future. Our study identified “limb pain” (D1) as a bridging symptom, connecting the somatic dyskinesia symptom cluster with the somatic-pain-related symptom cluster. Prior studies (52, 53) have reported that 40–65% of patients who recover from stroke experience pain in the extremities. This pain is mainly pathological, caused by lesions of the central somatosensory nervous system (54). Choi-Kwon et al. (55) previously reported that pain persists in patients recovering from stroke, leading to fatigue and the aggravation of depression, which constitutes a significant physical and psychological burden on patients, in turn affecting their daily life. Another previous study (56) showed that persistent pain during stroke recovery could cause a decrease in body function and an increase motor dysfunction (e.g., limb paralysis, muscle stiffness or spasticity) in the affected area, which can affect daily activities and quality of life. As previous studies (17, 19) have shown, bridge symptoms could represent as a focus for clinical symptom management interventions to sever the links between symptom clusters, achieve the deactivation of other symptom clusters connected to them, and improve the efficiency and precision of clinical interventions. It is recommended that healthcare professionals attend substantially to the symptoms of limb pain in patients recovering from stroke, and provide comprehensive assessment and intervention at an early stage, which should be based on the specific causes of limb pain, to develop a personalized treatment plan.

In addition, in the present study, we identified significant differences in prognosis and core symptoms between ischemic and hemorrhagic stroke. Therefore, in clinical practice, accurate diagnosis and differentiation of these two types of stroke, as well as corresponding treatment measures, are of great significance for improving patient prognosis. At the same time, conducting subgroup analysis to distinguish stroke types could also help to gain a deeper understanding of the characteristics and patterns of different types of stroke, providing a basis for developing more accurate treatment plans.

Network analysis is a statistical method that can be used to investigate the relationships between disease symptoms and effect the overall presentation of the disease. Network analysis can analyze multiple symptoms simultaneously, rather than viewing each symptom in isolation, which can help to reveal the interactions between symptoms. For example, Yang et al. (57) used network analysis methods to analyze the symptoms of internet addiction and suicidal tendencies among Chinese primary and secondary school students, finding that “requesting to extend online time” was the most important core symptom in the suicide internet addiction network model. By constructing a symptom network, core symptoms or symptom clusters, which may represent key targets for disease intervention, can be identified. In one prior study, through the construction of a symptom network, Kuang et al. (58) found that vomiting, fatigue, and sadness were important core symptoms elderly people with cancer. In addition, by analyzing symptom networks, the development and transformation of diseases can be predicted, providing a basis for early intervention. Zhu et al. (59) further used network analysis to explore the longitudinal relationship between depressive symptoms in middle-aged and elderly people in China. Research has found that “feeling fear” is a particularly important predictor. Network analysis has unparalleled advantages in revealing the complex connections between disease symptoms. However, this method also faces several challenges. Firstly, the results of network analysis are highly dependent on the accuracy and completeness of the input data. Any deviation or inaccuracy in this data could lead to misleading analysis results, resulting in incorrect conclusions. In addition, network analysis typically focuses on symptom relationships at specific time points or periods, which may limit its ability to capture the dynamic changes in symptom evolution over time.

## 5 Limitations

This study has several limitations which should be discussed. First, this was a cross-sectional investigation; therefore, we were only able to describe the relationships between symptoms, and could not make any causal inferences or predictions of symptoms. Future studies should continue to delve into this type of research by conducting longitudinal studies on symptom networks; exploring the developmental trajectories of symptom clusters, core symptoms, and bridging symptoms; and determining the causal relationships between symptoms. Second, the sample included in this study was small and was recruited from a single source; future studies should therefore recruit large samples from multiple centers. In addition, it should be noted that the research reference standards and symptom classification mainly refer to Chinese standards; further, some of the works cited in the article may not readily be available to readers from outside of China.

## 6 Conclusion

In this study, exploratory factor analysis identified five symptom clusters in patients recovering from stroke: the somatic activity disorder, mood disorders, cognitive–linguistic dysfunction, somatic-pain-related, and foot dysfunction symptom clusters. Among these, the somatic activity disorder symptom cluster represented the core symptom cluster. Further, symptom network analysis revealed “fatigue” (A5) and “pessimism about the future” (B3) as core symptoms in patients recovering from stroke, for which “Limb pain” (D1) was the bridge symptom. In clinical practice, we should prioritize interventions for these symptoms to weaken their ability to propagate through the network and reduce the symptom burden on patients.

## Data Availability

The data sets generated and analyzed during this study are available from the corresponding author upon reasonable request.
